# Delirium and Self-Reported Driving Behaviors and Outcomes After Critical Illness

**DOI:** 10.1001/jamanetworkopen.2025.31224

**Published:** 2025-09-10

**Authors:** Valerie Danesh, Brittany D. Work, Denise F. Chen, Julie Van, Matthew F. Mart, Sarah A. Welch, Mario Davidson, E. Wesley Ely, Shawniqua Williams Roberson, James C. Jackson, Leanne M. Boehm

**Affiliations:** 1 Baylor Scott & White Research Institute, Dallas, Texas; 2 Baylor College of Medicine, Houston, Texas; 3 Critical Illness, Brain Dysfunction, and Survivorship Center, Vanderbilt University Medical Center, Nashville, Tennessee; 4 Veterans Affairs Tennessee Valley Geriatric Research Education and Clinical Center, Nashville; 5 Department of Neurology, University of Washington, Seattle; 6 Division of Allergy, Pulmonary, and Critical Care Medicine, Vanderbilt University Medical Center, Nashville, Tennessee; 7 Department of Physical Medicine and Rehabilitation, Vanderbilt University Medical Center, Nashville, Tennessee; 8 Center for Musculoskeletal Research, Vanderbilt University Medical Center, Nashville, Tennessee; 9 Department of Biostatistics, Vanderbilt University Medical Center, Nashville, Tennessee; 10 Department of Medicine, Division of Allergy, Pulmonary, and Critical Care Medicine, Vanderbilt University Medical Center, Nashville, Tennessee; 11 Department of Neurology, Vanderbilt University Medical Center, Nashville, Tennessee; 12 Department of Medicine, Division of Pulmonary and Critical Care Medicine, Vanderbilt University Medical Center, Nashville, Tennessee; 13 School of Nursing, Vanderbilt University, Nashville, Tennessee

## Abstract

**Importance:**

Survivors of critical illness often have ongoing issues that affect functioning, including driving ability.

**Objective:**

To examine whether intensive care unit (ICU) delirium is independently associated with long-term changes in driving behaviors.

**Design, Setting, and Participants:**

This multicenter, longitudinal cohort study included 151 survivors of critical illness residing within 200 miles of Nashville, Tennessee. Survivors were surveyed 2 to 6 years after critical illness to assess the association between delirium duration and driving skills and behaviors (July 1, 2012, to April 30, 2013), and data were analyzed in 2014.

**Main Outcomes and Measures:**

The primary and secondary outcomes were self-rated driving skill (Driver Skill Inventory) and risky driving behaviors (Mini Driver Behavior Questionnaire), respectively. Exploratory outcomes included changes in driving decisions and driving-related attitudes.

**Results:**

Of 333 survivors from the original study, 151 (45.3%) were active drivers and agreed to participation at a median (range) of 3.7 (2.1-6.2) years after critical illness (median [IQR] age at enrollment, 59 [50-64] years; 87 [57.6%] male) with a median (IQR) delirium duration of 1 (0-4) day. Driving resumption occurred at a median (IQR) of 1.9 (0.8-6.0) months after ICU discharge. Delirium duration was not independently associated with self-rated driving skill or risky driving behavior measures (regression coefficients of −0.10 [95% CI, −1.13 to 0.93] for Driver Skill Inventory and–0.46 [95% CI, −1.97 to 1.06] for Mini Driver Behavior Questionnaire). Driving distances after critical illness were significantly lower than prehospitalization estimates (median [IQR], 60 [25-150] vs 105 [58-250] miles per week; *P* < .001). Driving skill (median [IQR], 34 [26-38] on a 48-point scale; *P* < .001) and safety orientation measures (median [IQR], 38 [35-46] on a 48-point scale; *P* < .001) were significantly lower upon return to driving. These decrements persisted among adults 65 years and older.

**Conclusions and Relevance:**

In this cohort study of survivors of critical illness, ICU delirium was not independently associated with long-term changes in driving behaviors after critical illness; survivors of critical illness self-reported significant decreases in transportation independence and driving skill after hospital discharge, independent of delirium duration during hospitalization. These results suggest that clinicians should consider transportation-related limitations relevant to short- and long-term care planning.

## Key Points

**Question:**

Is intensive care unit–acquired delirium associated with long-term changes in driving frequency or driving skill?

**Findings:**

In this cohort study of 151 survivors of critical illness, the recall of driving frequency, trip distances, and subjective ratings of driving skill were significantly lower after critical illness compared with before hospitalization. Delirium duration during critical illness was not associated with these changes.

**Meaning:**

Although survivors of critical illness recall changes in driving frequency, trip distances, and driving skill ratings during long-term recovery from critical illness, intensive care unit–acquired delirium is not an independent risk factor for these changes.

## Teaser

This cohort study of survivors of critical illness assesses whether the duration of delirium is associated with worse driving skill ratings and risk-prone driving behaviors after critical illness.

## Introduction

Survivors of critical illness are at risk of long-term cognitive impairment^1,2^ and functional disability.^3,4,5^ Delirium during critical illness represents a syndrome of fluctuating mental status, inattention, and disordered thinking. Delirium is an independent risk factor for cognitive and functional decline after critical illness.^1,6,7^ Cognitive decline is associated with many adverse sequelae, including reduced driving safety and worse driving performance,^8,9,10^ which can have important negative implications at both individual and public health levels. Drivers with cognitive impairment are at higher risk of motor vehicle–related crashes and injuries,^11,12,13^ whereas individuals who have stopped driving (voluntarily or involuntarily) experience loss of independence and community mobility.^14,15,16^

In many communities, the ability to drive is a key functional activity, profoundly influencing an individual’s lifestyle and mobility by dictating access to essential health care and medications and affecting opportunities for socialization.^16,17^ Unraveling the complex interplay between an individual’s ability to drive and their recovery after a critical illness is crucial because this knowledge not only reinforces realistic expectations but also guides the customization of critical illness recovery interventions, ultimately enhancing autonomy.

Despite the pivotal nature of driving to both independence and community mobility, it remains grossly understudied as a long-term outcome of critical illness.^18^ Most studies^19^ evaluating long-term outcomes after critical illness rely on standardized assessment batteries that examine physical, behavioral, and cognitive function as well as employment status and health care use but fail to incorporate driving assessment. To address this knowledge gap, we examined changes in driving patterns, driving skills, and safety outcomes in a longitudinal follow-up study of participants from a prospective cohort of critical illness survivors enrolled from 2 medical centers. Because delirium during critical illness is a significant risk factor for new-onset and persistent cognitive impairment and disability,^1,20^ we explored the effects of delirium on driving-related outcomes. Given the high prevalence of delirium occurrence in the main study, for this substudy we hypothesized that the duration of delirium is associated with worse driving skill ratings and risk-prone driving behaviors after critical illness.

## Methods

### Design

Data for this point-prevalence cohort study nested within the multicenter prospective Bringing to Light the Risk Factors and Incidence of Neuropsychological Dysfunction in ICU Survivors (BRAIN-ICU) cohort were collected from participants enrolled in a longitudinal, prospective cohort assessing potentially modifiable risk factors of long-term cognitive impairment after critical illness.^1^ Informed consent for this driving substudy was obtained orally from each participant at the time of the long-term follow-up interview. All study protocols, consent documents, and questionnaires for the BRAIN-ICU study, including this follow-up study on driving after critical illness, were approved by the Vanderbilt University institutional review board. The study followed the Strengthening the Reporting of Observational Studies in Epidemiology (STROBE) reporting guideline.^23^

### Participants

**Figure 1.**
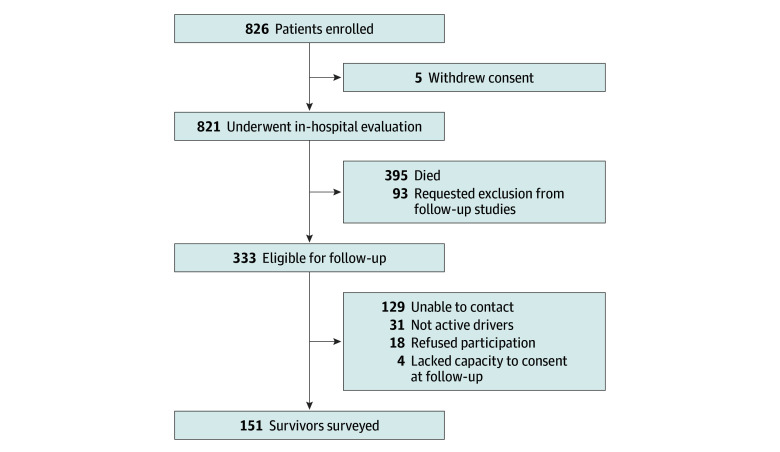
Study Flowchart

Participants had normal cognition before critical illness assessed by the Informant Questionnaire on Cognitive Decline in the Elderly (IQCODE) for global cognitive impairment as rated by a close relative during the index hospitalization.^21,22^ The original BRAIN-ICU cohort study enrolled adults (aged >18 years) admitted to an intensive care unit (ICU) with acute respiratory failure or shock. Additional inclusion criteria for this nested follow-up study were driving resumption after critical illness and driving activity within the previous 12 months among participants who survived to 2 years after their index hospitalization. We enrolled 826 medical and surgical ICU patients in the initial BRAIN-ICU study (January 1, 2007, to December 31, 2010), of whom 333 participants were alive at 2 years after hospital discharge and eligible for contact to participate (Figure 1). Among participants who were alive and eligible for this nested study, 35 participants (10.5%) reported long-term driving disability or did not have capacity to consent, 31 (9.3%) declined to participate, and 133 (39.9%) were lost to follow-up.

### Data Collection and Measures

Demographics, comorbidity burden (Charlson Comorbidity Index), acuity (Acute Physiology and Chronic Health Evaluation II), delirium onset and duration (Confusion Assessment Method for the ICU), daily level of consciousness (Richmond Agitation Sedation Scale), and cognitive status before critical illness (IQCODE) were assessed during the index hospitalization. Participant race and ethnicity classifications were self-reported by the participant at the time of enrollment in the main study. We collected data on race and ethnicity to explore their role in ICU outcomes. Cognitive status (Mini-Mental Status Examination) was assessed at hospital discharge.

The nested telephone survey to assess participant driving frequency, driving trip characteristics, and subjective ratings of driving skill occurred at 2 to 6 years after hospital discharge. The 45-item driving survey (eMethods in Supplement 1) included measures drawn from the Mini Driver Behavior Questionnaire (Mini-DBQ),^24^ Driver Skill Inventory (DSI),^25,30^ and the Driving Habits Questionnaire (DHQ).^26^ The Mini-DBQ and DSI were used as primary and secondary outcome measures, whereas the DHQ served as an exploratory outcome. The DHQ was modified to include questions about participants’ driving frequency and the characteristics of their driving trips, both before their index hospitalization and at the time of assessment. Driving measures were collected for 3 periods. Participants provided retrospective ratings for 2 periods: prehospitalization (time 1) and the period immediately after return to driving after critical illness (time 2). Importantly, these recall-based assessments were collected at 2 to 6 years after hospitalization during the long-term follow-up telephone interview, and time 2 was defined as the first month of return to driving. Current driving measures (time 3) were collected in real time during the same interview, using the standard administration procedures for these measures.

### Statistical Analysis

Participant characteristics and assessment measures were summarized descriptively using means (SDs) or medians (IQRs). Driving distances before and after critical illness were examined using the Wilcoxon signed rank test. Association between delirium duration and self-rated driving skill was examined using quantile regression with prespecified covariates: age, comorbidity burden, acuity during index hospitalization, patient-reported driving skill, and cognitive status before (using the IQCODE) and after critical illness (using the Mini-Mental State Examination). For quantile and linear regression, coefficients represent the estimated difference in medians or means. Linear tail-restricted cubic splines with 3 knots were used to relax the linearity assumption for age, patient-reported driving skill ratings before critical illness, delirium duration, and severity of illness. The association between delirium duration and risky driving was also examined by fitting an ordinary least-squares regression model with the same a priori covariates but excluded subjective driving skill ratings prior to critical illness. Missing cognitive assessments prior to critical illness (IQCODE) for 39 participants were imputed using age-stratified population norms. A 2-sided *P* < .05 was considered statistically significant. Because both delirium and dementia are more common in older adults, we also examined baseline characteristics in groups stratified by older age (≥65 years); however, no formal statistical comparison between groups was completed. Analysis was completed between January and March 2014.

## Results

This nested follow-up study includes 151 survivors of critical illness (median [IQR] age at enrollment, 59 [50-64] years; 87 [57.6%] male and 64 [42.4%] female; 18 [11.9%] African American or Black and 133 [88.1%] White) who were actively driving 2 to 6 years after critical illness, with older participants (aged ≥65 years) reporting shorter delays of a median (IQR) of 1.2 (0.5-3.0) months compared with younger participants (aged <65 years) reporting driving resumption after a median (IQR) of 2.0 (1.0-6.0) months.

Participants 65 years or older had shorter hospitalizations but were otherwise similar to those younger than 65 years. Median (IQR) delirium duration was 1 (0-4) day. The median (IQR) Mini-Mental State Examination score at the time of discharge was 27 (23-29). The median (range) time between index hospitalization and study driving assessment was 3.7 (2.1-6.2) years (Table 1). Driving status, driving safety, and driving outcomes before and after critical illness are reported in Table 2.

**Table 1.** Self-Reported Demographics and Clinical Characteristics

Characteristic	No. (%) of participants^a^		
	Aged 18-64 y (n = 113)	Aged ≥65 y (n = 38)	Overall (N = 151)
Sex			
Female	50 (44.2)	14 (36.8)	64 (42.4)
Male	63 (55.8)	24 (63.2)	87 (57.6)
Race			
African American or Black	18 (15.9)	0	18 (11.9)
White	95 (84.1)	38 (100.0)	133 (88.1)
Charlson Comorbidity Index, median (IQR)	2 (0-3)	1 (1-3)	2 (0-3)
APACHE II score, median (IQR)	20 (14-25)	20 (15-24)	20 (14-25)
ICU length of stay, median (IQR), d^b^	4.8 (2.1-10.9)	3.9 (1.7-6.9)	4.7 (2.0-9.9)
Hospital length of stay, median (IQR), d^c^	10.9 (6.1-19.0)	7.7 (4.1-12.0)	9.2 (6.0-17.1)
Cognitive status (IQCODE) before ICU stay, median (IQR)	3.00 (3.00-3.00)	3.00 (3.00-3.06)	3.00 (3.00-3.00)
Cognitive status (MMSE) at discharge, median (IQR)	26 (23-29)	27 (20-28)	27 (23-29)
Delirium duration, median (IQR), d	NA	NA	1 (0-4)
Hospital discharge destination			
Home	80 (70.8)	25 (65.8)	105 (69.5)
Short-term rehabilitation	27 (23.9)	8 (21.1)	35 (23.2)
Nursing home or LTAC	3 (2.7)	5 (13.2)	8 (5.3)
Time since hospital discharge, median (IQR), y	3.7 (3.0-4.3)	3.7 (3.1-4.3)	3.7 (3.0-4.3)

Abbreviations: APACHE II, Acute Physiology and Chronic Health Evaluation II; ICU, intensive care unit; IQCODE, Informant Questionnaire on Cognitive Decline in the Elderly; LTAC, long-term acute care facility; MMSE, Mini-Mental State Examination; NA, not applicable.

^a^ Unless otherwise indicated.

^b^ Hospital length of stay and ICU length of stay are as counted during the 14-day study period.

^c^ MMSE scores were obtained at discharge for 147 of the 151 participants.

**Table 2.** Driving Status, Driving Safety, and Driving Outcomes After Critical Illness

Driving characteristic	No. (%) of participants^a^		
	Before critical illness (n = 113)	After critical illness (n = 38)	Overall (N = 151)
Driving status			
Former driver	1 (0.9)	3 (7.9)	4 (2.6)
Currently driving	112 (99.1)	35 (92.1)	147 (97.4)
Requested to limit driving	1 (0.9)	3 (7.9)	15 (9.9)
Subjective driving skill			
Poor or fair	7 (6.2)	1 (2.6)	8 (5.3)
Average	10 (8.8)	8 (21.1)	18 (11.9)
Good	43 (38.1)	22 (57.9)	65 (43.0)
Excellent	53 (46.9)	7 (18.4)	60 (39.7)
Driving frequency, median (IQR), d/wk	7 (5-7)	5 (3-7)	NA
Driving distance, median (IQR), m/wk	105 (58-250)	60 (25-150)	NA
Driving Skills Inventory, mean (SD)^b^			
Predicting traffic situations	3.27 (0.78)	2.34 (1.14)	NA
Changing lanes in heavy traffic	3.25 (0.69)	2.67 (1.03)	NA
Controlling the vehicle	3.48 (0.68)	2.80 (1.02)	NA
Passing another car	3.37 (0.74)	2.69 (0.98)	NA
Driving through a nonsignalized crossing	3.38 (0.72)	3.01 (0.99)	NA
Concentration	3.19 (0.66)	2.70 (1.01)	NA
Wayfinding	2.91 (0.94)	2.50 (1.17)	NA
Vehicle crashes in the past year as the vehicle driver			
None	NA	129 (85.4)	NA
≥1	NA	22 (14.6)	NA
2	NA	3 (2.0)	NA
Traffic violations			
None	NA	144 (95.4)	NA
≥1	NA	7 (4.6)	NA

Abbreviation: NA, not applicable.

^a^ Unless otherwise indicated.

^b^ Driving Skills Inventory is scored on a 5-point Likert scale, with higher scores indicating higher skill ratings.

### Association of Delirium With Driving Outcomes

Delirium duration was not an independent risk factor for total driving skill score at long-term follow-up (regression coefficient, −0.10; 95% CI, −1.13 to 0.93; *P* = .86). Delirium duration was also not independently associated with self-reported risky driving (regression coefficient, −0.46; 95% CI, −1.97 to 1.06; *P* = .42).

### Driving Status

**Figure 2.**
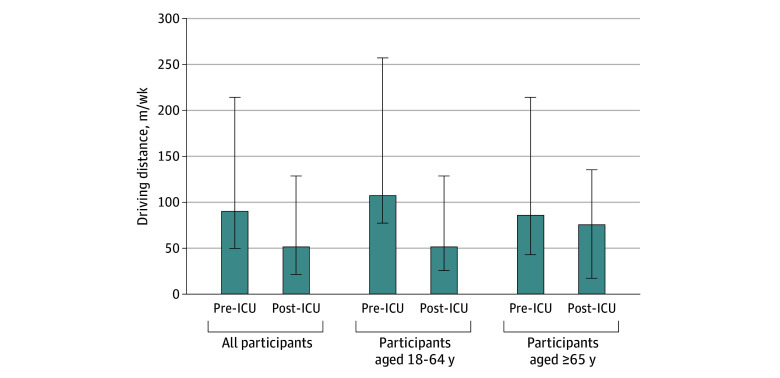
Driving Distance Before and After Critical Illness by Age Group Error bars indicate SDs. ICU indicates intensive care unit.

Among the 333 participants who were approached but did not participate in the driving follow-up assessment, 31 were identified as nonactive drivers and excluded, and 4 were excluded because of capacity to provide consent due to presumed incapacity to drive. This subset reflects a driving cessation prevalence of at least 10.5%. Driving distances (miles per week) before and after critical illness are presented in Figure 2. Driving distances after critical illness were significantly lower compared with distances reported prior to critical illness (median [IQR], 60 [25-150] vs 105 [58-250] miles per week; *P* < .001). This difference was more pronounced in the group 65 years or younger (median [IQR], 60 [30-150] vs 125 [90-300] miles; *P* < .001) compared with participants 65 years or older (median [IQR], 88 [20-158] vs 100 [50-250]; *P* < .001). Driving frequency also decreased, with participants driving on fewer days after critical illness (median [IQR], 5 [3-7] days per week) compared with driving frequency before critical illness (median [range], 7 [5-7] days per week). The median (IQR) duration to return to driving after critical illness was 1.9 (0.8-6.0) months. Some participants reported experiencing anxiety and/or simply did not feel physically strong enough to drive while recovering from critical illness.

### Driving Safety

A total of 125 participants (82.8%) rated their driving quality as good or excellent despite lower scores after critical illness (median [IQR], 38 [35-46] vs 34 [26-38] on a 48-point scale; *P* < .001). Significant differences between scores before and after critical illness remained upon stratification by age (eFigure in Supplement 1). Participants younger than 65 years more commonly reported driving improvement (median [IQR], 39 [35-46] vs 34 [23-38]; *P* < .001). Driving skill ratings prior to critical illness were independently associated with DSI score at follow-up. Among participants, 15 (9.9%) reported that they had been advised to stop or limit their driving due to safety concerns in the past year. Furthermore, 22 participants (14.6%) experienced one or more driving-related accidents within the year after hospitalization for a critical illness.

Among active drivers, 66 (44.9%) reported using self-regulation strategies, specifically by avoiding certain driving situations (eTable in Supplement 1). The most avoided driving situations were driving at night in the rain (69 [45.7%]) and driving at night (54 [35.1%]). Driving scenarios associated with reliance on executive function included avoiding driving through busy intersections (13 [8.6%]), driving on freeways (22 [14.6%]), and driving routes with high traffic density (23 [15.2%]). Most participants who reported changes in their driving behavior characterized these adjustments as newly adopted strategies implemented after their experiences with critical illness. The frequency of participants reporting challenges in particular driving contexts appeared comparable between older and younger age groups.

### Driving Outcomes

Participants reported risky driving outcomes in 3 categories: errors, lapses, and violations. The mean (SD) item score for the entire study sample was 0.6 (1.1); all items scored between 0 (never engages in behavior) and 1 (hardly ever engages in behavior) with the exception of item 6 (forgetting where you left your car in a parking lot; mean [SD], 1.2 [1.2]). The lapses subscore was the greatest (mean [SD], 0.9 [0.7]) followed by the violations (mean [SD], 0.5 [0.6]) and errors (mean [SD], 0.5 [0.6]) subscores.

The mean (SD) total score for participants 65 years or older (4.1 [3.3]) tended to be better than the mean (SD) total score for participants younger than 65 years (5.2 [3.4]). The mean (SD) subscores for driving violations also were better in participants 65 years or older (0.84 [1.36] and 2.3 [1.8], respectively) compared with those younger than 65 years (1.66 [2.06] and 2.7 [2.0], respectively). The mean (SD) subscore for lapses tended to be worse in participants 65 years or older (1.00 [1.20]) compared with those younger than 65 years (0.88 [1.04]). Controlling for other covariates, higher IQCODE scores (cognitive status rated much worse after critical illness) and higher acuity (Acute Physiology and Chronic Health Evaluation II scores on admission) were associated with higher rates of self-reported risky driving.

## Discussion

In this nested point-prevalence cohort study of long-term driving outcomes among 151 survivors of critical illness, we found that driving frequency, distance, and skills were significantly lower 2 or more years after critical illness compared with before hospitalization, with older adults reporting persistent decrements in driving skill. Interestingly, although delirium is independently associated with long-term cognitive impairment after the ICU, we did not find an association between delirium and driving outcomes. Driving a personal vehicle is a key but often overlooked component of recovery after hospitalization that requires physical, behavioral, and cognitive skills. Driving status changes can be direct and indirect reflections of physical, behavioral, and cognitive sequelae of critical illness and should be incorporated as important patient-centered outcome in ongoing research on critical illness survivorship. Delirium may not have been recognized as an independent risk factor for driving changes in this cohort due to a survivorship bias of low prevalence of delirium among participants eligible for follow-up (ie, those alive and driving 2-6 years after critical illness hospitalization).

Approximately 25% of survivors of critical illness experience cognitive deficits commensurate with mild Alzheimer disease or moderate traumatic brain injury at 12 months after discharge,^1^ with mechanical ventilatory support and ICU-acquired delirium^2^ representing the most prominent risk factors. Drivers with cognitive impairment are at higher risk of vehicle crashes and injuries^11,12^ and impaired wayfinding.^27^ Comparable to driving studies specific to Alzheimer disease and traumatic brain injury,^8,9,10^ survivors of critical illness report reduced concentration, feelings of unpreparedness, decreased mental alertness, and impaired spatial awareness as barriers for driving.^28^ Additionally, physical ability limited by weakness, fatigue, and ongoing recovery were limiting factors in driving resumption.^28^ Although these deficits are well-recognized characteristics of postintensive care syndrome, there are few investigations on returning to driving for this population.^28^ These findings underscore the importance of understanding the driving-related behavior patterns after critical illness.

Driving status in critical illness recovery is an emerging outcome measure. To date, small-scale self-report studies in the US, UK, and Australia have described that a subset of survivors of critical illness driving prior to critical illness do not resume driving (driving cessation) in the year after critical illness.^18^ Driving cessation rates in the early recovery period range from 31% to 50% during the first 3 months^29,30,31^ and 22% to 24% at 6 months^29,32^ and are 16% at 12 months.^29^ These insights suggest that the changes in driving attributed to recovery from critical illness are more sudden onset than those attributed to aging, which are more gradual and progressive. With a median follow-up time of 3.7 years in this BRAIN-ICU cohort and expanded measures of driving frequency, trip distance, and subjective ratings of driving skill, our observations extend long-range insights into functional outcomes after critical illness.

A major finding of this study was a significantly decreased weekly driving distance compared with miles driven prior to critical illness. Our participants most frequently reported avoiding nighttime driving, driving in the rain, and parallel parking. This finding is concerning given the impact of driving on community mobility, the loss of which is associated with depression and other conditions.^16^ Driving safety concerns may also undermine a survivor’s ability to return to work or reliance on others as well as access to health care. When survivors of critical illness change their driving status, family members likely play a crucial role in meeting transportation needs while managing their own responsibilities, potentially contributing to caregiver burden.^33^ As with evaluations of return to work after critical illness,^34^ the implications of driving status affect both the individual and broader household or community units. Assessing employment status and other social determinants of health is warranted to examine the association with driving status and driving safety after critical illness, particularly in the setting of emerging technology enabling autonomous driving. The inclusion of the broader household membership and care partners is warranted in future research and guidelines.

The changes in subjective driving quality scores, while numerically limited, likely reflect meaningful shifts in self-perception of ability after critical illness. Importantly, these changes align with qualitative themes in similar cohorts of survivors of critical illness, namely, uncertainty about when it is safe to resume driving and concern about rare but serious risks (eg, collisions).^30^ Overall, these modest score differences illustrate a broader issue that there is no clear threshold for what constitutes a clinically meaningful change in driving decisions after critical illness. This ambiguity reinforces the need for clinical decision support tools tailored to functional outcomes such as driving, for which binary go or no-go decisions include nuanced and subjective self-assessments.

### Strengths and Limitations

The key strength of this study is the use of validated driving measures (eg, driving skill, risky driving) and precritical illness cognitive status (eg, family-reported IQCODE) to report driving trip distance and frequency before critical illness for the first time. However, the study also has limitations. There are limitations associated with the study design and measurement. For example, collecting patient-reported driving measures at a median of 3.7 years after critical illness could be viewed as a limitation because of recall bias, despite offering a broader view of long-term driving status, including self-reports of infrequent traffic incidents reported in this sample. Very low event rates for traffic incidents are not surprising given that studies evaluating traffic incident rates require large samples and treat collisions as Poisson-distributed events. Other work has evaluated bias using longer recall periods for health indicators, including 5-year recall periods.^35^

Additionally, this nested study reflects a cohort with modest exposure to prolonged delirium with a median (IQR) of 1 (0-4) days, thus potentially contributing some selection bias for associations with delirium. Furthermore, given that driving measures were not collected from individuals reporting driving cessation at the time of contact for this study (≥2 years after hospital discharge), the reasons for complete driving cessation in this population are important to understand and should be evaluated in future studies. Self-report measures of driving status and driving safety^36,37^ in the context of validated neuropsychological assessments are a first step in defining the epidemiology of driving behaviors after critical illness, with self-report considered reliable for describing disability.^38^ Future study designs can advance measurement from self-report to naturalistic kinematic driving assessments.

## Conclusions

In this cohort study of long-term driving outcomes, survivors of critical illness reported persistent impairments in driving skill, manifesting as changes in driving distances and driving behaviors, but these changes are not associated with delirium in the hospital. In critical illness recovery, the risk factors for these changes are important and remain unknown. Additional large-scale, longitudinal studies are needed to identify modifiable risk factors and trajectories to design and test safety interventions relevant to recovery and rehabilitation after critical illness. Additionally, advancing knowledge in driving decision-making and risk factors for changes in driving are needed for individual resource planning and could be relevant to community-level needs (eg, urban planning), particularly in communities with increasing percentages of older adults.

## Notes

Supplement 1. **eMethods.** Supplemental Text **eTable.** Driving Avoidance Characteristics After Critical Illness **eFigure.** Reported Changes in Driving Skills Over Time by Age Group
eReferences

Supplement 2.
Data Sharing Statement
