# Supplementary material for: Delirium and Self-Reported Driving Behaviors and Outcomes After Critical Illness
Source: JAMA Netw Open. 2025 Sep 10;8(9):e2531224. doi: 10.1001/jamanetworkopen.2025.31224 (PMC12423849; doi:10.1001/jamanetworkopen.2025.31224)
Supplement: Supplement 1. — eMethods. Supplemental Text eTable. Driving Avoidance Characteristics After Critical Illness eFigure. Reported Changes in Driving Skills Over Time by Age Group eReferences [file jamanetwopen-e2531224-s001.pdf]

Supplemental Online Content

Danesh V, Work BD, Chen DF, et al. Delirium and self-reported driving behaviors and outcomes after critical illness. *JAMA Netw Open*. 2025;8(9):e2531224. doi:10.1001/jamanetworkopen.2025.31224

eMethods. Supplemental Text
eTable. Driving Avoidance Characteristics After Critical Illness
eFigure. Reported Changes in Driving Skills Over Time by Age Group
eReferences

This supplemental material has been provided by the authors to give readers additional information about their work.

© 2025 Danesh et al. *JAMA Network Open*

eMethods. Supplemental Text

This supplementary material has been provided by the authors to give readers additional information about their work on 1) data collection, population, and settings; 2) measures; 3) statistical analysis; and 4) methodology references.

Data collection, population, and settings

The initial enrollment occurred at ICU admission between March 2007 and May 2010, and the follow-up surveys were conducted in 2012. Analyses were conducted between March 2014 and January 2022. Race and ethnicity data were collected and reported to support the evaluation of the study's representativeness and to allow transparent reporting of the demographic characteristics of the cohort.

Measures

This appendix provides additional detail on the Post-ICU Driving Outcomes Survey administered for this study. The *Driving Habits* section was derived from items 1-11 and 25-28 of the Driving Habits Questionnaire (DHQ) and evaluates current driving status, driving exposure (average number of days and miles driven in a week), and self-reported number of crashes and traffic citations. The *Driving Skills* section was adapted from the Driver Skill Inventory (DSI), which assesses strengths and weaknesses regarding specific driving scenarios and measures an individual's "safety orientation," i.e. an individual's opinion of himself or herself as a skilled or safe driver[1]. The 12 items in Section 3 were selected from the "perceptual motor skills" component of the DSI, which has demonstrated value for predicting accident risk[1]. Participants rated their driving strengths and weaknesses on a 5-point Likert scale ranging from 0 (definitely weak) to 4 (definitely strong) at three time points: before ICU hospitalization, in the

© 2025 Danesh et al. *JAMA Network Open*

initial months upon returning to driving following ICU discharge, and at the time the questionnaire was administered (2-5 years post-ICU discharge). For each time point, the total DSI score ranged from 0 (definitely weak on all skills) to 48 (definitely strong on all skills).

The *Driving Avoidance* section included 10 questions adapted from Baldock et al.[2], which were adapted from items 17-24 of the DHQ[3]. These items evaluate avoidance of difficult driving situations as a measure of driving self-regulation[2]. The *Risky Driving* section included 8 items from the Mini-DBQ[4], which measures self-reported frequency of aberrant driving behavior categorized as violations, errors, and lapses based on the driver's intention behind and outcome of the behavior[4,5]. The Mini-DBQ is a validated, abridged adaption of the Manchester Driver Behavior Questionnaire (DBQ); one recent meta-analysis demonstrated that the violation and error items of the DBQ are predictors of self-reported accidents[6]. Responses for Section 5 were scored on a 5-point Likert scale ranging from 0 (never engages in behavior) to 5 (nearly always engages in behavior); the minimum and maximum total score for this section were 0 and 40, respectively.

Prompts and phrasing and response type for each item are provided:

**Question**	**Response Type**
**Driving Habits**	
1. Do you currently drive?	Y/N
2. Why did you stop driving?	Open-ended
3. When is the last time you drove?	Date
4. Has anyone suggested over the past year that you limit your driving or stop driving?	Y/N

© 2025 Danesh et al. *JAMA Network Open*

Question	Response Type
5. How would you rate the quality of your current (or most recent) driving?	5 point Likert
6. Before your ICU stay, how many miles per week did you drive in an average week?	Number
7. After your ICU stay?	Number
8. Before your ICU stay, how many days per week did you drive in an average week?	Number
9. After your ICU stay?	Number
10. After your ICU stay, how many accidents have you been involved in, as the driver, over the past year? Please tell me the number of all accidents, and whether or not you were at fault.	Number
11. After your ICU stay, how many times have you been pulled over by the police, regardless of whether you received a ticket?	Number
12. After your ICU stay, how many times in the past year have you received a traffic ticket (other than a parking ticket) where you were found to be guilty, regardless of whether or not you think you were at fault?	Number
**Driving Skills**	
Prompt: Now, we will ask you some questions about the strong and weak components of your driving. Please think about the strengths and weaknesses of your driving before your ICU stay, immediately after your ICU stay, and now. Please answer the following questions on a scale of 0 to 4 (0 = Definitely weak, 1=Weak, 2 = Neither weak nor strong, 3 = Strong, 4 = Definitely strong)	
1. Performance in a risky situation (e.g., emergency maneuvers in unexpected risky situation).	5-point Likert
2. Predicting traffic situations ahead (i.e., anticipating what will happen).	5-point Likert
3. Knowing how to act in particular traffic situations (i.e., choosing the most appropriate behavior in the situation concerned).	5-point Likert
4. Changing lanes in heavy traffic.	5-point Likert
5. Reacting quickly to the environment around you while driving.	5-point Likert
6. Controlling the vehicle.	5-point Likert
7. Making a hill start on a steep incline.	5-point Likert

Question	Response Type
8. Passing another car.	5-point Likert
9. Parallel parking in reverse into a narrow gap.	5-point Likert
10. Driving through a non-signalized crossing.	5-point Likert
11. Finding my way in roads which are not familiar to me.	5-point Likert
12. Concentrating on driving when distracted (e.g. by a passenger or a phone call).	5-point Likert
**Driving Avoidance**	
Prompt: We are interested in whether or not you avoid certain driving conditions. Please answer yes or no to the following questions.	
1. Do you avoid driving in the rain?	Y/N
2. Do you avoid driving alone?	Y/N
3. Do you avoid parallel parking?	Y/N
4. Do you avoid making right turns?	Y/N
5. Do you avoid driving through busy intersections?	Y/N
6. Do you avoid driving on freeways?	Y/N
7. Do you avoid driving on high traffic roads?	Y/N
8. Do you avoid driving during rush hour?	Y/N
9. Do you avoid driving at night?	Y/N
10. Do you avoid driving at night in the rain?	Y/N
**Mini-DBQ**	
Prompt: For the next set of questions, please indicate, on a scale from 0 to 5 (see below), how often you experience these situations.<br>0: Never, 1: Hardly ever, 2: Occasionally, 3: Quite often, 4: Frequently, 5: Nearly all the time.	
1. Tail-gating the car ahead of you as a signal for that driver to go faster or get out of your way.	6-point Likert
2. Deliberately disregarding the speed limits late at night or very early in the morning.	6-point Likert

© 2025 Danesh et al. *JAMA Network Open*

Question	Response Type
3. Getting involved in unofficial "races" with other car drivers (just "in his mind alone" e.g. racing off from the traffic lights to be the quickest).	6-point Likert
4. Turning right onto a main road into the path of an oncoming vehicle you had not seen, or whose speed you had misjudged.	6-point Likert
5. Failing to notice that a pedestrian is starting to cross the road.	6-point Likert
6. Forgetting where you left your car in a multi-level parking lot.	6-point Likert
7. Getting into the wrong lane at a traffic circle or when approaching an intersection.	6-point Likert
8. Failing to read traffic signs correctly and exiting from a traffic circle on the wrong road.	6-point Likert

eTable. Driving Avoidance Characteristics After Critical Illness

**Driving Characteristics**	**After Critical Illness (*n* = 151),** n (%)
Do you avoid driving in the rain? (yes)	45 (29.8)
Do you avoid driving alone? (yes)	12 (7.9)
Do you avoid parallel parking? (yes)	51 (33.8)
Do you avoid making right turns? (yes)	1 (0.7)
Do you avoid driving through busy intersections? (yes)	13 (8.6)
Do you avoid driving on freeways? (yes)	22 (14.6)
Do you avoid driving on high traffic roads? (yes)	23 (15.2)
Do you avoid driving during rush hour? (yes)	39 (25.8)
Do you avoid driving at night? (yes)	53 (35.1)
Do you avoid driving at night in the rain? (yes)	69 (45.7)

© 2025 Danesh et al. *JAMA Network Open*

eFigure. Reported Changes in Driving Skills Over Time by Age Group

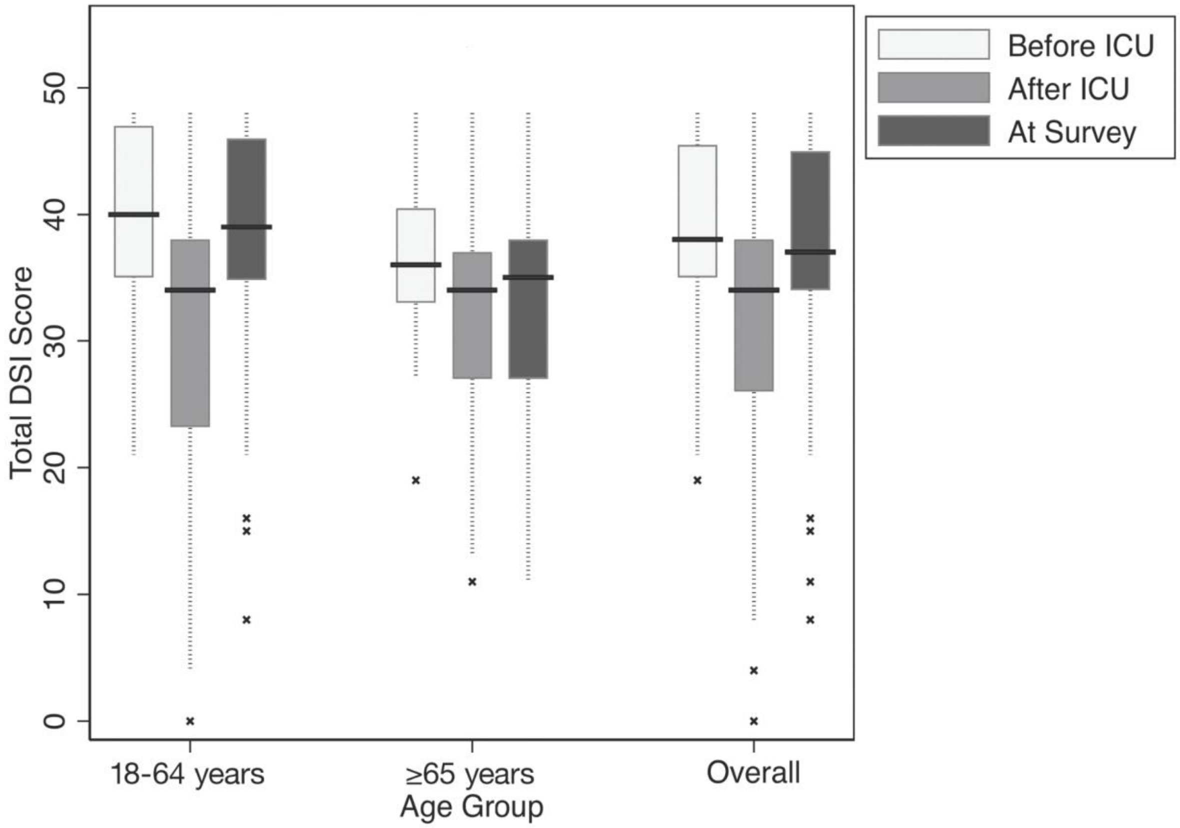

© 2025 Danesh et al. *JAMA Network Open*

eReferences

1. Lajunen T, Özkan T. Self-report instruments and methods. In: Porter B, ed. *Handbook of Traffic Psychology.* Vol 1. 1 ed. London: Academic Press; 2011:43-59.
2. Baldock MRJ, Mathias JL, McLean AJ, Berndt A. Self-regulation of driving and its relationship to driving ability among older adults. *Accident Analysis & Prevention.* 2006;38(5):1038-1045.
3. Owsley C, Stalvey B, Wells J, Sloane ME. Older drivers and cataract: Driving habits and crash risk. *J Gerontol A Biol Sci Med Sci.* 1999;54(4):M203-M211.
4. Martinussen LM, Lajunen T, Moller M, Ozkan T. Short and user-friendly: The development and validation of the Mini-DBQ. *Accident Analysis & Prevention.* 2013;50:1259-1265.
5. Reason J. Human error: Models and management. *BMJ.* 2000;320(7237):768-770.
6. de Winter JC, Dodou D. The Driver Behaviour Questionnaire as a predictor of accidents: a meta-analysis. *Journal of Safety Research.* 2010;41(6):463-470.

© 2025 Danesh et al. *JAMA Network Open*
