# Supplementary material for: Delirium and Self-Reported Driving Behaviors and Outcomes After Critical Illness
Source: JAMA Netw Open. 2025 Sep 10;8(9):e2531224. doi: 10.1001/jamanetworkopen.2025.31224 (PMC12423849; doi:10.1001/jamanetworkopen.2025.31224)
Supplement: Supplement 2. — Data Sharing Statement [file jamanetwopen-e2531224-s002.pdf]

Data Sharing Statement

Danesh. Delirium and Self-Reported Driving Behaviors and Outcomes After Critical Illness. *JAMA Netw Open*. Published September 10, 2025. doi:10.1001/jamanetworkopen.2025.31224

Data
Data available: No

Additional Information
Explanation for why data not available: De-identified data will be made available upon reasonable request
